# Comparative Removal of Lead and Nickel Ions onto Nanofibrous Sheet of Activated Polyacrylonitrile in Batch Adsorption and Application of Conventional Kinetic and Isotherm Models

**DOI:** 10.3390/membranes11010010

**Published:** 2020-12-23

**Authors:** Muhammad Tahir Amin, Abdulrahman Ali Alazba, Muhammad Shafiq

**Affiliations:** 1 Alamoudi Water Research Chair, King Saud University, P.O. Box 2460, Riyadh 11451, Saudi Arabia; alazba@ksu.edu.sa (A.A.A.); msrana@ksu.edu.sa (M.S.); 2 Department of Environmental Sciences, COMSATS University Islamabad, Abbottabad Campus, University Road, Abbottabad 22060, Pakistan; 3 Agricultural Engineering Department, King Saud University, P.O. Box 2460, Riyadh 11451, Saudi Arabia

**Keywords:** adsorption capacity, electrospinning, Langmuir and Freundlich, PANmod, physisorption, pseudo-second-order

## Abstract

We investigated the adsorption of lead (Pb^2+^) and nickel (Ni^2+^) ions by electrospun membranes of polyacrylonitrile (PAN) nanofiber activated with NaHCO_3_ (PANmod). Analysis by Fourier-transform infrared spectrometry (FTIR), field emission scanning electron microscopy (FE-SEM), and energy dispersive X-ray spectroscopy (EDX) validated the functionalization of PAN nanofibers with NaHCO_3_, and the successful agglomeration of Pb^2+^ and Ni^2+^ onto PANmod. After a rapid uptake of the heavy metal ions (15 min), the equilibrium contact time was attained (60 min) following a linear increase of both adsorption capacity and removal efficiency. PANmod showed a better affinity for Ni^2+^ than Pb^2+^. The adsorption on PANmod was best described by the pseudo-second-order kinetic model for both studied models, supporting chemisorption. By varying the solution pH from 2.0 to 9.0, we found that the adsorption capacity followed an increasing trend, reaching a maximum at the pH of 7.0. Despite increasing adsorption capacities, the removal efficiency of both heavy metal ions exhibited a decreasing trend with increase in initial concentrations. The amount of PANmod directly affects the removal efficiency, with 0.7 and 0.2 g being the optimum dose for maximum uptake of Pb^2+^ and Ni^2+^, respectively. The Langmuir model fitted well the Pb^2+^ adsorption data suggesting monolayer adsorption, and the Freundlich model perfectly fitted the Ni^2+^ adsorption data, indicating heterogeneous adsorption. The estimated values of the mean free energy of adsorption in the D–R isotherm indicated a physical adsorption of both heavy metal ions into the surface of the PANmod.

## 1. Introduction

Rapid industrial development and urbanization introduced large amount of waste effluents into various environmental compartments [1]. Those effluents contain large amounts of toxic metals/metalloids, drastically polluting the ecosystem. Among toxic metals, nickel (Ni^2+^) and lead (Pb^2+^) abound in industrial wastewaters produced during mining, battery and machinery manufacturing, mineral processing, metal coating, and cable sheathing, as well as by steam electric power plants and stainless steel alloys, plating and tanning industries [2,3]. Elevated levels of the non-biodegradable Ni^2+^ and Pb^2+^ ions in freshwater sources pose a significant threat to animal and human health, because they accumulate in living tissues through the food chain [4]. Exposure to metals/metalloids may induce severe deterioration in human health including memory loss, cardiovascular disease, abdominal pain, weakness, chronic bronchitis and cancer of the lung [5]. Therefore, in pursuance of a pollution-free ecosystem, these toxic metals/metalloids should be eliminated from industrial wastewater before it is discharged into freshwater resources.

Various wastewater treatment techniques such as reverse osmosis, ion exchange, chemical precipitation, electrodialysis, chemical coagulation, solvent extraction and adsorption processes have been employed to remove metals/metalloids from industrial wastewater [6,7,8,9,10]. However, most of these methods are either inefficient or costly [11]. Adsorption technology is emerging as the best technology for wastewater purification due to its simplicity of design, low cost, ease in operation, reduced output of harmful sludge, insensitivity to pollutants, reduced energy consumption, high effectivity and environment compatibility [12]. The adsorption efficiency is directly linked to the materials used in the adsorption process. The most important characteristics of the adsorbent are higher surface area, porosity and the chemical structure [13,14]. In addition, the surface polarity and chemical composition of the adsorbent can affect the attraction forces between adsorbent and adsorbate [15]. Therefore, selection of an appropriate adsorbent is of critical importance. Polymer nanofiber membrane-based adsorbents prepared by the electrospinning technique have been widely used for metals/metalloids removal from wastewater, due to their unique properties of high porosity, specific pattern of fine pores, and larger surface area [16,17]. Polyacrylonitrile (PAN) is a synthetic semi-crystalline organic polymer which does not melt under normal condition, but does at 300 °C [18]. Furthermore, PAN can be electrospun due to its optimum stability, great chemical resistance, high solubility, and great mechanical strength [19,20,21]. Because of these unique properties, PAN is a potential candidate as a nano-fibrous adsorbent for wastewater treatment [22]. The adsorption efficiency of membranes made of PAN can be increased by adding various types of nanomaterials to the PAN/DMF solution before electrospinning, or by applying certain chemical treatments after fabrication to increase its functionality [20,23]. The main objective of treating nano-fibrous membranes with chemical treatments or cross-linking of the nano fibrous membrane is that, under specific conditions, the functional groups on the surface of nanofibers are modified into an appropriate form, depending on the nature of the adsorbate which may be involved in chemical reactions with complexation agents of metal ions [24]. Researchers have been using chemically treated nanofiber membranes such as PAN/EDTA [19], and PAN/NaOH-NaHCO_3_ [25], for removal of dyes and metals/metalloids ions since the last decade. It has been assumed that the functionalization of the PAN nanofibers surface is the most important factor for improving the adsorption efficiency of adsorbents used in the elimination of metal ions.

The electrospun sheet of a PAN nanofiber treated with NaHCO_3_ (PANmod) has not yet been tested for adsorption of heavy metal ions from aqueous systems. This endeavor is the focus of the current study. Our main objective was to functionalize and characterize the PANmod, and determine its feasibility as an efficient adsorbent for the removal of Pb^2+^ and Ni^2+^ from aqueous solutions. Furthermore, we evaluated adsorption performance employing conventional kinetics and isotherm models.

## 2. Materials and Methods

### 2.1. Fabrication and Characterization of the PANmod

We prepared a 10% (*w*/*v*) solution of polymer PAN ((C_3_H_3_N)_n_; Sigma-Aldrich, St. Louis, MO, USA) and solvent dimethylformamide (DMF, C_3_H_7_NO; Sigma-Aldrich, 99.8%), which was homogenized at room temperature using a magnetic stirrer (IKA Werke GmbH, Staufen, Germany) for approximately 12 h. The resulting mixture was injected to an electrospinning device (NanoNC Electrospray system, Seoul, Korea) at a flow rate of 1 mL h^−1^ using a 5 mL glass syringe. The distance between the needle tip and the grounded metal collector was set at 15 cm and the electrospinning device was operated at high voltage (15 kV) supplied by a high voltage generator (Model: ESN-HV30). The resulting sheet was prepared in a rectangular shape (5 × 7 cm^2^), dipped in 150 mL of 15% NaOH solution (60 min, 60 °C), and then washed several times with distilled water. Next, to neutralize the excess amount of base, the sheet was immersed in 100 mL of 1.0 M HCl solution. Subsequently, the hydrolyzed sheet was treated with 100 mL of 10% NaHCO_3_ solution for 6 h, to prepare the final product. The fabricated PANmod sheet was cut into small pieces, to be used as an adsorbent for heavy metal ions. Its chemical groups and surface morphology were examined by Fourier-transform infrared spectrometry (FTIR) (Vertex 70 Bruker, Karlsruhe, Germany), field emission scanning electron microscopy (FE-SEM) (JSM-7600 JEOL, Tokyo, Japan) and energy dispersive X-ray spectroscopy (EDX) (Inca software installed inside FE-SEM).

### 2.2. Experimental Setup and Analysis

Stock solutions of Pb^2+^ (1000 mg L^−1^) and Ni^2+^ (1000 mg L^−1^) were prepared from analytical reagent grade precursors, lead nitrate (Pb(NO_3_)_2_; Tianjin Benchmark Chemical Reagent Co., Ltd. Tianjin, China) and nickel nitrate hexahyhydrate (Ni(NO_3_)_2_·6H_2_O; Tianjin Benchmark Chemical Reagent Co., Ltd. Tianjin, China), respectively. 1000 mg of each precursor was dissolved in 1.0 L of deionized water. Each stock solution was further diluted, to achieve the initial concentrations of Pb^2+^ or Ni^2+^ required in different batch tests; the solution pH was maintained at various fixed values (2–9) by using either diluted sodium hydroxide (NaOH) or diluted hydrochloric acid (HCl). 50 mL of metal solution with different initial concentrations (5–50 mg L^−1^) were agitated for fixed time intervals (5–300 min) in a temperature-controlled shaker (Wise Cube orbital, Wisd. ThermoStable IS-20; Daihan Scientific Co. Ltd., Wonju, South Korea), adding the amount of PANmod to achieve the required dose (0.1–0.9 g) in each batch trial. After shaking them in conical flasks (100 mL) for a predetermined time, the resultant mixture was filtered through 0.45 μm filter paper (Whatman™) and the residual concentration of metal was determined using flame atomic absorption spectrometry (FAAS, Thermo Scientific, ICE 3000 Series, Cambridge, United Kingdom). The amount of metal uptake at time *t*, that is the adsorption capacity (*q_t_*, mg g^−1^) and the effective of the adsorption of metal ions into PANmod were determined using the following equations:

(1) $$Adsorption\;capacity=\left(\frac{\left(C_{0}-C_{t}\right)\cdot V}{m}\right)$$

(2) $$Removal\;\left(\%\right)=\left(\frac{C_{0}-e}{C_{0}}\right)\;\times 100,$$

where *C*_o_ (mg L^−1^) is the concentration of either Pb^2+^ or Ni^2+^ before the batch adsorption and *C_e_* (mg L^−1^) is the equilibrium concentration of either Pb^2+^ or Ni^2+^. *C_t_* (mg L^−1^) is the concentration of either metal ions after sorption for a specified contact time and corresponds to *q_t_* (mg g^−1^) for evaluating the kinetics of the adsorption process. *V* (L) is the volume of the metal ions’ solution and *m* (g) is the mass of the PANmod. For the isotherm models, the equilibrium adsorption capacity, *q_e_* (mg g^−1^), was evaluated from Equation (1), corresponding to the equilibrium concentration of metal ions measured at equilibrium contact time *C_e_* (mg L^−1^).

## 3. Results and Discussion

### 3.1. PANmod Characteristcs

#### 3.1.1. FTIR Analysis

**Figure 1:**
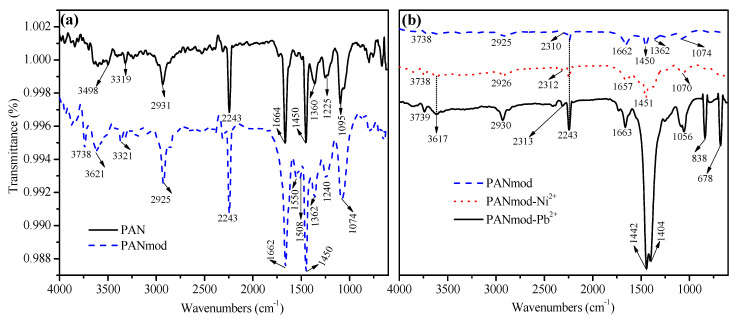
Fourier-transform infrared spectrometry (FTIR) analysis of (**a**) the polyacrylonitrile (PAN) nanofibers and PAN nanofiber activated with NaHCO_3_ (PANmod) and (**b**) PANmod, PANmod-Ni^2+^ and PANmod-Pb^2+^.

To investigate modification in the functional groups and surface chemistry of PAN nanofibers, pristine and Ni^2+^ and Pb^2+^ loaded PANmod samples were analyzed by the FTIR technique; the acquired spectra are presented in Figure 1. The sharp peak at 1095 cm^−1^ is ascribed as stretching vibrations of the C−O group, whereas the peaks at 1225 and 1360 cm^−1^ are designated as vibrations of C−O−C and C−OH, respectively (Figure 1a). Likewise, sharp peaks appearing at 1450, 3319 and 3498 cm^−1^ reveal the stretching vibrations of −CH_2_−, O−H, and N−H groups [26]. The peaks appearing at 1664, 2243, and 2931 cm^−1^ correspond to stretching vibrations of the C=O, C−N, and C-H groups, respectively [27]. After the PAN nanofiber was treated with NaHCO_3_, the spectral peaks appearing at 1095, 1664 and 2931 cm^−1^ shifted to the lower wave number positions 1074, 1662, and 2925 cm^−1^, respectively, while other peaks such as 1225, 1360, and 3319 cm^−1^ shifted to the higher wave number positions 1240, 1362, and 3321 cm^−1^, respectively [28].

In addition, new low intensity peaks noted at 1508 and 1550 cm^−1^ were ascribed to the aromatic N-H secondary amine group [28]; other new peaks appearing around 3621 and 3738 cm^−1^ were ascribed to stretching vibrations of O−H group [29]. These spectral changes indicate the interactions between PAN nanofibers and NaHCO_3_ functionalization by the formation of polar interactions between the functional groups of both components. Polar interactions of the functional groups might help in the adsorption of divalent heavy metal ions.

The vibrational spectra of Ni^2+^ and Pb^2+^ loaded PANmod are shown in Figure 1b. In Ni^2+^ loaded PANmod, the 1070 cm^−1^ spectral peak is ascribed to C−O bonds. The vibrational spectral peaks at 1451, 1657, 2312, 2926, and 3738 cm^−1^ are ascribed to the −CH_2_−, C=O, C−H, and O−H groups, respectively [27]. In the Pb^2+^ loaded PANmod sample spectra, some peaks were shifted and some new peaks were observed, when compared to PANmod. The shifted spectral features are the 1056, 1404, 1442, 1663, 2313, 2930, and 3739 cm^−1^ peaks. Moreover, the two new peak positions around 678, and 838 cm^−1^ were ascribed to metal (M= Ni^2+^, Pb^2+^) and oxygen (M−O or M−O−M) interactions [30]. FTIR spectral analysis revealed a successful adsorption of Ni^2+^ and Pb^2+^ into the PANmod surface.

#### 3.1.2. FE-SEM and EDX Analysis

**Figure 2:**
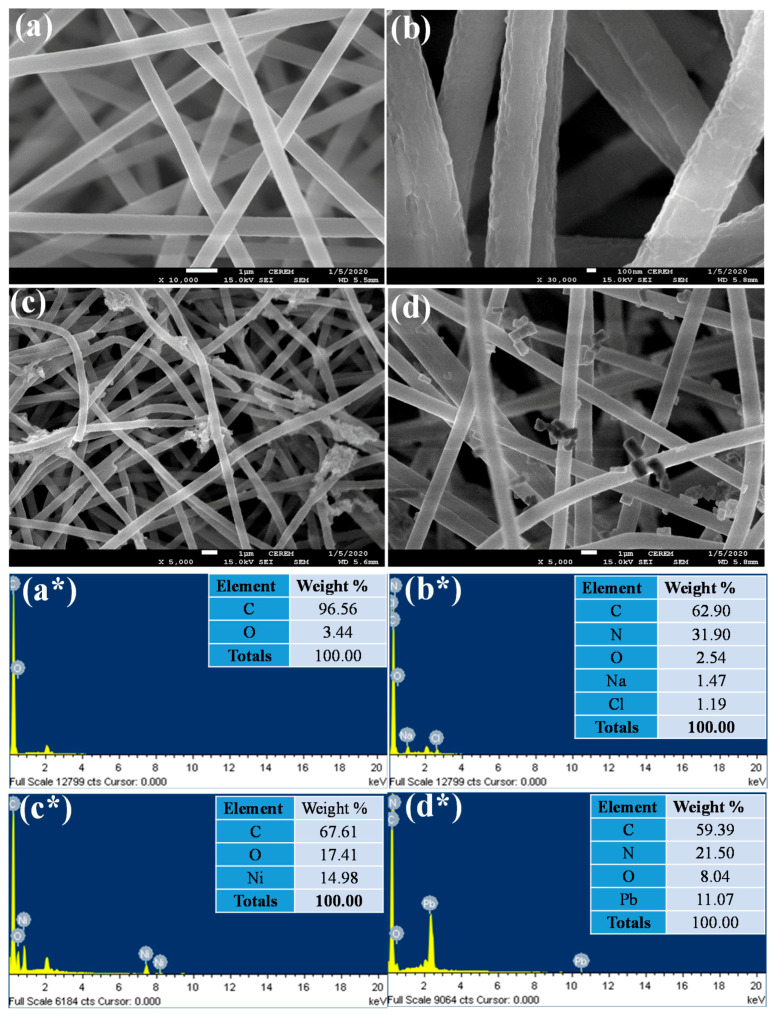
Field emission scanning electron microscopy (FE-SEM) and energy dispersive X-ray spectroscopy (EDX) spectra of PAN nanofibers (**a**,a*), PANmod (**b**,b*), PANmod- Ni^2+^ (**c**,c*), and PANmod- Pb^2+^ (**d**,d*).

We investigated by FE-SEM and EDX the changes in elemental composition and morphological features of the pure PAN nanofibers, as well as pristine and Ni^2+^ and Pb^2+^ loaded PANmod (Figure 2). Very smooth, cylindrical, randomly arranged, and beads-free nanofibers of pure PAN are clearly seen in Figure 2a. Their EDX analysis (Figure 2a*) reveal that the C and O content in these nanofibers are 96.56% and 3.44%, respectively. The rough and porous surfaces of the PANmod nanofibers are clearly shown by the FE-SEM image in Figure 2b; the EDX analysis (Figure 2b*) of the same nanofibers reveals their successful functionalization. Additionally, the elemental composition of the PANmod show that the contents of C, N, O, Na, and Cl are 62.90, 31.90, 2.54, 1.47, and 1.19%, respectively.

Furthermore, the agglomeration of Ni^2+^ onto nanofibers can clearly be seen in the FE-SEM image of the post-adsorption Ni^2+^ loaded PANmod samples (Figure 2c). The EDX analysis of Ni^2+^ loaded PANmod (Figure 2c*) shows an increase in C (67.61%) and O contents (17.41%), compared with pristine nanofibers; 14.98% of Ni^2+^ are also observed, suggesting the adsorption of Ni^2+^ on these nanofibers. Similarly, the FE-SEM image of the post-adsorption Pb^2+^ loaded PANmod reveals very small particles attached to the nanofibers, probably Pb^2+^ (Figure 2d). Interestingly, the EDX spectrogram of the PANmod-Pb^2+^ (Figure 2d*) shows a reduction in C (59.39%) and N contents (21.50%), and an increase in O content (8.04%), 11.07% of Pb^2+^ suggests the successful adsorption of Pb^2+^ on PANmod-Pb^2+^. This analysis reveals that the functionalization of the PAN nanofibers with NaHCO_3_ is quite beneficial for the adsorption of Ni^2+^ and Pb^2+^ metal ions.

### 3.2. Influence of the Contact Time and Fitting of Kinetic Models to the Adsorption Data of Both Heavy Metal Ions

**Figure 3:**
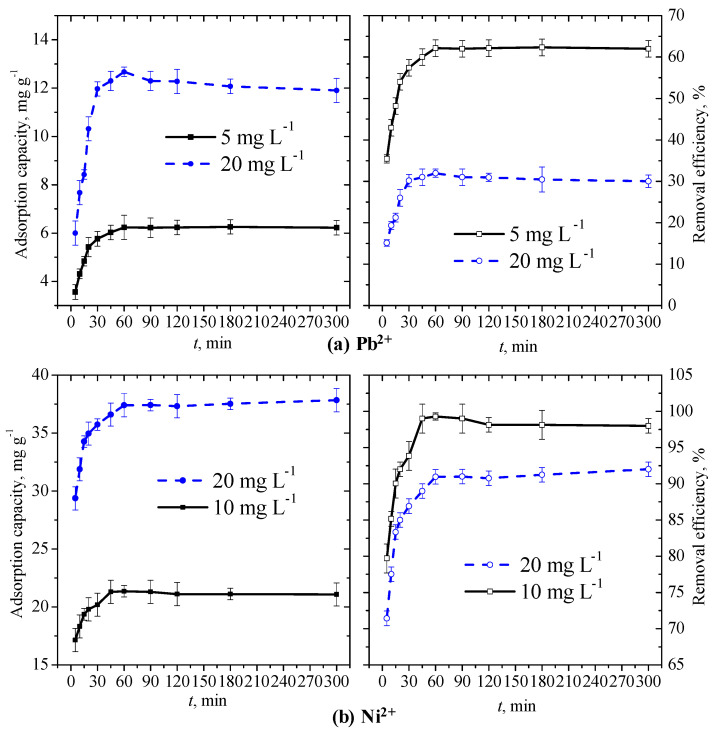
Variations with time of the adsorption capacity and removal efficiency of (**a**) Pb^2+^ and (**b**) Ni^2+^ by PANmod.

We estimated the equilibrium contact time for both heavy metal ions by performing time-series batch experiments; Figure 3 shows the variations in both the adsorption capacity and removal efficiency by PANmod at different initial concentrations of Pb^2+^ and Ni^2+^. The solution pH was maintained at 5.0 and 7.0 for Pb^2+^ and Ni^2+^, respectively, by using a fixed amount (0.5 g) of PANmod. Our observations clearly demonstrate a very rapid uptake of both heavy metal ions at any tested initial concentration, during immediate contact with PANmod. In the first 15 min of contact time, one can see almost 70–80% (Figure 3a) and 90% (Figure 3b) of the maximum removal efficiency of, respectively, Pb^2+^ and Ni^2+^ by PANmod. This could be the result of adsorbed metal ions accumulation on the unused surface and free active sites of the PANmod. After rapid uptake of the adsorbate, a nearly linear increase can be observed in both the adsorption capacity and the removal efficiency, equilibrium being attained at about 60 min since contact started. An additional 4 h of contact yielded insignificant (*p =* 0.01) effects on the adsorption performance (Figure 3), due to the saturation of the absorption sites of the PANmod. For 20 mg L^−1^ initial metal concentration, the maximum adsorption capacity at 60 min was 12.7 and 37.4 mg g^−1^ with corresponding removal efficiency of 32 and 92% for Pb^2+^ and Ni^2+^, respectively. This indicates a dominant effect and better efficiency of the absorption performance of the PANmod for Ni^2+^, compared to Pb^2+^.

Conventional kinetic models were applied to the data collected using the heavy metal ions adsorption on PANmod; and these included the pseudo-first-order (PFO, Equation (3)), pseudo-second-order (PSO, Equation (4)), intraparticle diffusion of Weber and Morris (ID-WM, Equation (5)), and Elovich (Equation (6)) kinetic models.

(3) $$q_{t}=q_{e}\;\left(1-\exp\left(-k_{1}t\right)\right)$$

(4) $$q_{t}=\frac{{q_{e}}^{2}k_{2}\cdot t}{q_{e}k_{2}\cdot t+1}$$

(5) $$q_{t}=K_{ip}t^{1/2}+C$$

(6) $$q_{t}=\frac{1}{\beta }\ln(1+\alpha \beta t)$$

**Figure 4:**
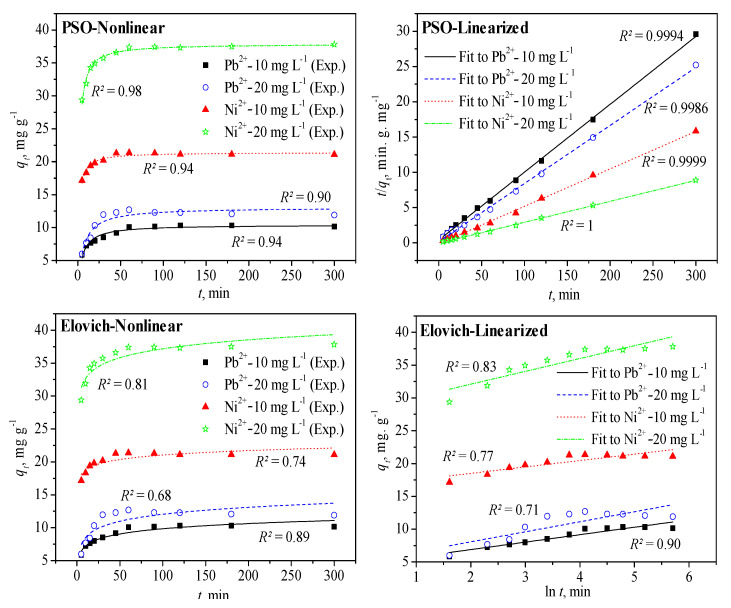
Fitting of the pseudo-second-order (PSO) and Elovich kinetic models to the adsorption data of Pb^2+^ and Ni^2+^ on PANmod at different initial concentrations.

In the above equations, *q_t_* (mg g^−1^) is the amount of adsorbed Pb^2+^ or Ni^2+^, while *q_e_* (mg g^−1^) is the equilibrium adsorption capacity of the PANmod for metal ions, *k*_1_ (min^−1^) is the rate constant for the PFO kinetic model and *k*_2_ (mg g^−1^ min^−1^) for the PSO kinetic model. The estimated rate constant in the PSO kinetic model is further used to evaluate the initial adsorption rate *h* (*k_2_ q_e_*^2^; mg g^−1^ min^−1^). For the ID-WM kinetic model, the rate constant is expressed by *K_ip_* (mg g^−1^ min^1/2^) and the boundary-layer thickness by *C* (mg g^−1^). In the Elovich kinetic model, *α* (mg g^−1^ min^−1^) is the initial adsorption rate constant and *β* (g mg^−1^) expresses the chemisorption activation energy. Figure 4 shows the PSO and Elovich kinetic models fitting of the adsorption data for Pb^2+^ and Ni^2+^ with initial concentrations of 10 and 20 mg L^−1^; both the nonlinear and linearized approaches were used. The solution pH was maintained at 5 ± 0.2 and 7 ± 0.3 for Pb^2+^ and Ni^2+^, respectively, and the dose of PANmod was 0.5 g. Table 1 presents the values of the related parameters in all nonlinear models as estimated using the OriginPro 8.5 software at different initial concentrations of both metal ions.

The PSO kinetic model linearized fitting yielded a perfect fit of the model to the adsorption of both heavy metal ions on PANmod at the initial concentrations tested (10 and 20 mg L^−1^), with a coefficient of determination (*R^2^*) close to unity (1.0). The nonlinear fitting of the PSO kinetic model yielded also a good fitting of the adsorption data with *R^2^* in the range 0.90–0.98, with Ni^2+^ presenting a relatively good fit compared to the adsorption data of Pb^2+^, at 20 mg L^−1^. For the Elovich kinetic model, no significant difference between the *R^2^* for nonlinear and linearized fitting was observed, with the linearize fitting yielding somewhat better results than the nonlinear approach. In addition, at 20 mg L^−1^, the models fitted better the adsorption data for Ni^2+^ than that of the Pb^2+^, as was the case in the PSO kinetic model, but at an initial concentration of 10 mg L^−1^, adsorption data of Pb^2+^ was better described by the Elovich model that that of Ni^2+^.

The results presented in Table 1 for nonlinear fitting with conventional kinetic models show that, compared to the other models, the PSO model described the best the adsorption of both studied models on PANmod, based on the calculated *R^2^* values (0.9–0.99). The only exception was found for 20 mg L^−1^ in the case of Pb^2+^where the PFO kinetic model proved to be slightly better than the PSO model. This has helped to demonstrate that the chemisorption is the governing mechanism of adsorption involving valence forces due to exchange or sharing of electrons between the metal ions and the PANmod. The estimated adsorption capacities matched well with the experimental values for both PFO and PSO kinetic models with PSO yielding a slightly better agreement than the PFO kinetic model at the equilibrium contact time (60 min), as shown in Table 1. The rate constant in the PFO kinetic model was not affected by changing the initial metal concentration of both heavy metal ions (except at 20 mg L^−1^ of Pb^2+^) with higher values for Ni^2+^, compared to Pb^2+^(Table 1).

For the PSO kinetic model, the rate constant reduced to almost half for both heavy metal ions as their initial concentration was doubled, i.e., 10 to 20 mg L^−1^, with Ni^2+^ showing higher *k_2_* values than Pb^2+^ at equal initial concentrations. The initial adsorption rate was much higher for Ni^2+^ compared with Pb^2+^ (*h*, Table 1), and increased with initial metal concentrations increase for both types of ions. The ID-WM kinetic model did not fit the adsorption data well for neither of the ion types (*R^2^* ranged between 0.46 and 0.64, at initial concentrations of 5 to 20 mg L^−1^). Both rate constant and boundary-layer thickness increased as the initial metal concentrations increased, exhibiting higher values for Ni^2+^ compared to Pb^2+^ at equal initial concentrations, with the exception of *K_ip_* at 10 mg L^−1^(Table 1). The Elovich kinetic model fitted reasonably the adsorption data on PANmod for both types of ions, for all initial concentrations, as reflected by the *R^2^* values in Table 1.

### 3.3. Influence of the Solution pH, Initial Ion Concentration, and Dose of Adsorbent on Adsorption Performance and Regeneration of PANmod

**Figure 5:**
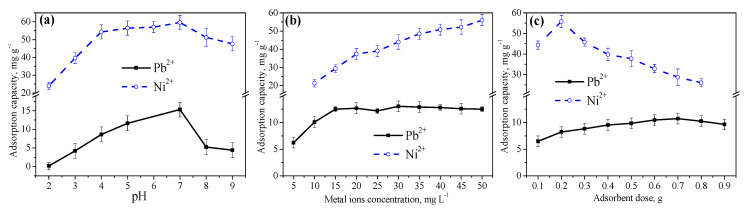
The adsorption capacity of Pb^2+^ and Ni^2+^ vs. (**a**) solution pH, (**b**) initial metal’s concentration, and (**c**) dose of PANmod.

The variation in absorption capacity and removal of heavy metal ions obtained by changing the pH (2–9) of the metal solution is shown in Figure 5a. The bath test was conducted at the equilibrium contact time (60 min) using the adsorbent dose of 0.5 g of PANmod and 10 and 20 mg L^−1^ of Pb^2+^ and Ni^2+^, respectively, as initial ion concentrations. The excess H^+^ created a competition of absorb on the PANmod surface, with divalent Pb^2+^ and Ni^2+^ [31,32] resulting a very low uptake for both ion types at low pH values. The maximum adsorption capacity of nearly 15 and 60 mg g^−1^ for Pb^2+^ and Ni^2+^, respectively, was found at a solution pH of 7.0, following a trend of increasing adsorption capacity with increasing pH due to the lowered amount of H^+^ and reduced competition for divalent Pb^2+^ and Ni^2+^ to absorb on the PANmod surface [32,33]. Subsequently, a decrease in metal uptake indicated the pH of 7.0 as optimum value, as shown in Figure 5a. The percentage removal, after following an increasing trend for both ion types, decreased from 73% to 21% and from 91% to 73% for Pb^2+^ and Ni^2+^, respectively, as the solution pH increased further from 7.0 to 9.0.

The absorption potential of the PANmod was optimized for the initial concentration of heavy metal ions; the observed changes are depicted in Figure 5b. An equilibrium contact time of 60 min was chosen by fixing the dose of PANmod at 0.5 g while a solution pH of 5.0 and 7.0 was maintained for Pb^2+^ and Ni^2+^, respectively. The PANmod uptake of Pb^2+^ increased linearly as the initial metal concentration increased from 5 to 20 mg L^−1^; a further increase in the initial metal concentration did not affect in the adsorption capacity, which remained almost constant at the maximum value of approximately 13 mg g^−1^, as shown in Figure 5b. The Ni^2+^ adsorption, however, continued to increase linearly with its initial concentration increase, attaining the maximum adsorption capacity of 56 mg g^−1^ at the maximum used value of the initial concentration (50 mg L^−1^). A concentrated solution with high initial concentration of adsorbate will result in quick adsorption and increased adsorption capacity of a fixed amount of the adsorbent (PANmod in this study) due to strong driving forces between divalent metal ions and active adsorbent sites [34,35]. On the other hand, a decreasing trend in the removal efficiency of both types of ions was seen, due to the limited capacity of the fixed amount of PANmod (0.5 g) and, hence, offering a reduced number of active sites to an increased metal concentration. Finally, a 50% difference different in percentage removal was seen when the initial concentration of Pb^2+^ and Ni^2+^ increased from 5 and 10 to 50 mg L^−1^, respectively (Figure 5b).

Figure 5c shows variation in the adsorption capacity due to changes in the amount of PANmod (0.1–0.9 g). The equilibrium contact time was 60 min for the batch test, and the solution pH was maintained at 5.0 and 7.0, with a fixed initial concentration of 10 and 20 mg L^−1^, for Pb^2+^ and Ni^2+^, respectively. The optimum dose of PANmod, corresponding to a maximum uptake of Pb^2+^ and Ni^2+^, was found to be 0.7 and 0.2 g, respectively. A decrease in the adsorption capacity when the adsorbent dose further increased is associated with the high surface area of the PANmod available to absorb a fixed amount of metal ions, that is 10 and 20 mg L^−1^ for Pb^2+^ and Ni^2+^, respectively. The amount of PANmod has a direct effect on the removal efficiency which increased almost linearly and reached ~90% and about 100% for Pb^2+^ and Ni^2+^, respectively, at the maximum tested value of 0.9 g of PANmod. The adsorption capacities of both metal ions, as estimated in the current study, using the PANmod is compared with other similar adsorbents in previous studies (Table 2).

Batch experiments were conducted aiming at the regeneration of the PANmod, considering its reusability and adsorption capacities were estimated for both heavy metal ions in different sets of desorption experiments at different initial solution pH and temperature. FE-SEM analysis already revealed a strong bonding and affinity between the PANmod surface and the absorbed metal ions (Pb^2+^ and Ni^2+^), so diluted hydrochloric acid (0.1 M HCl) was used as eluent in addition to a mild heat treatment inside an oven for 24 h. The amount of desorbed metal ions was high at low solution pH (2–5) with average desorption rate reaching almost 100% while the amounts of both metal ions also decreased as temperature increased.

### 3.4. Application of Isotherm Models to Adsorption Data of Pb^2+^ and Ni^2+^

The equilibrium data was evaluated with the commonly employed isotherm models and the biosorption process was analyzed. Table 3 shows the original (nonlinear) and linearized mathematical expressions of the two-parameter isotherm models; the Sips and Redlich–Peterson (R–P), among the three-parameter isotherm models are expressed by:

(7) $$q_{e}=\frac{q_{m}K_{S}{C_{e}}^{n_{S}}}{1+K_{S}{C_{e}}^{n_{S}}}$$

(8) $$q_{e}=\frac{q_{m}K_{S}{C_{e}}^{n_{S}}}{1+K_{S}{C_{e}}^{n_{S}}}$$

Parameters *C_e_* (mg L^−1^) and *q_e_* (mg g^−1^), which are used in all expressions of Table 3, represent the residual metal ion concentration in the solution and the corresponding equilibrium adsorption capacity. *q_m_* (mg g^−1^) denotes the estimated maximum adsorption capacity in the Langmuir, Freundlich, Dubinin–Radushkevich (D–R) models (Table 4) and the Sips isotherm model (Equation (7)). The dimensionless separation factor *R_L_* (Table 3) in the Langmuir model is used to describe the shape of the isotherm [42] and is dependent on the Langmuir constant, *K_L_* (L mg^−1^), which shows the affinity between the heavy metal ions and the PANmod surface. In the Freundlich model, the favorability of the adsorption system is shown by the dimensionless *n* in the range 2–10 [43], while the model constant is expressed as *K_F_* ((mg g^−1^)(L mg^−1^)^1/n^). The mean free energy of adsorption *E* (kJ mol^−1^, Table 3) in the D–R model is relevant to the model constant *K_DR_* (mol kJ^–1^)^2^ and is used to describe whether the adsorption system is physical (*E* < 8 kJ mol^−1^) or chemical (*E* > 8 kJ mol^−1^). *T* (Kelvin) and *R* (8.314 J mol^−1^·K^−1^) are used in the D–R and Temkin models and represent the absolute temperature and universal gas constant, respectively. The terms *n_H_* and *k_H_* are the exponent and constant in the Halsey isotherm, respectively, while both *A_HJ_* and *B_HJ_* are constants evaluated in the Harkins–Jura (H–J) model. The heat of adsorption and the binding constant at equilibrium in the Temkin model are expressed by *H_ads_* (kJ mol^−1^) and *K_T_* (L mg^−1^), respectively (Table 3).

**Figure 6:**
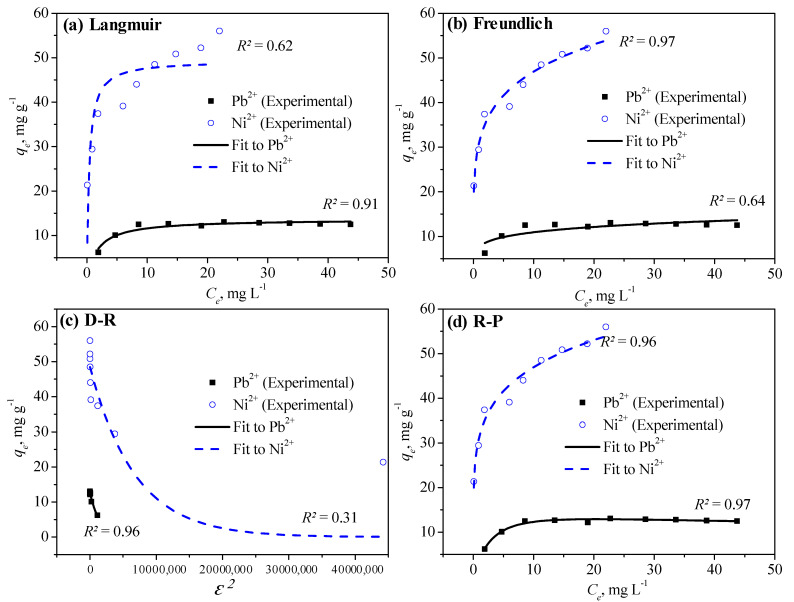
Nonlinear and linearized fitting of (**a**) Langmuir, (**b**) Freundlich, (**c**) Dubinin–Radushkevich (D–R), and (**d**) Redlich–Peterson (R–P) to the adsorption data of Pb^2+^ and Ni^2+^ onto PANmod.

The constant of the Sips model, *K_S_* (L g^−1^), is related to the energy of adsorption, while the degree of heterogeneity is expressed by the dimensionless number (*n_S_*) in Equation (7). Both *α* (L mg^−1^) and dimensionless *β* (0–1) are parameters used in R–P isotherm with the model’s constant being expressed by *K_RP_* (L g^−1^) in Equation (8). Table 4 lists the values of the parameters mentioned above for all the isotherm models evaluated, by using the slope and intercept values in the respective linearized plots; OriginPro 8.5 and CurveExpert Professional software were used for the nonlinear approach. The suitability of the nonlinear two-parameter and three-parameter isotherms to the experimental data of both heavy metal ions is shown in Figure 6 for an equilibrium contact time of 60 min, as described earlier. We analyzed the adsorption data for batch tests performed at 30 °C by using 0.5 g of PANmod; the solution pH was maintained at 5 ± 0.2 and 7 ± 0.2 for Pb^2+^ and Ni^2+^, respectively.

As shown in Figure 6a, the Langmuir model poorly fitted the Ni^2+^ adsorption; the same result was obtained by applying the Freundlich model to Pb^2+^ adsorption data (Figure 6b). This is reflected by low *R^2^* for both nonlinear and linearized fittings of the Ni^2+^ and Pb^2+^ adsorption in the Langmuir and Freundlich isotherm models, respectively (Table 4). The theoretical adsorption capacities were in good agreement with the experimental values for both the Langmuir and Freundlich isotherm models for Pb^2+^, but a ~10% disagreement between the experimental and calculated values was observed for Ni^2+^ in both models. The values of the separation factor in the Langmuir model, 0 < *R_L_* < 1, and the dimensionless factor *n* of 2–10 in the Freundlich model indicated that both isotherms fit well the adsorption of Pb^2+^ and Ni^2+^ by both the nonlinear as well as the linearized approach (Table 4). Considerably higher values of the model constants for both Langmuir and Freundlich isotherms were obtained for Ni^2+^, in comparison to Pb^2+^; the *K_L_* values of 2.66 and 11.89 L mg^−1^ (Table 4) show the better affinity between the PANmod surface and Ni^2+^, compared with Pb^2+^, respectively. On the other hand, high *R^2^* values and close agreement of the theoretical adsorption capacities with the experimental values for the Langmuir model suggest a monolayer adsorption of Pb^2+^ on the PANmod surface.

Figure 6c shows that the D–R isotherm fitted the Ni^2+^ adsorption data poorly, which is further reflected by the very low *R^2^* value 0.31 (Table 4); however, the model described well the Pb^2+^ adsorption data with both the linear and nonlinear approaches; the calculated adsorption capacities are in close agreement with those of the experimental values. The estimated values of the mean free energy of adsorption, *E* < 8 kJ mol^−1^ clearly reflect a physical adsorption of both types of heavy metal ions on the PANmod surface (Table 4). Based on the observed *R^2^* values, the Temkin isotherm also represented better the adsorption data of Ni^2+^ compared to Pb^2+^, as shown in Table 4. This reflects the heterogeneous adsorption [44] of Ni^2+^ with uniform distribution of binding energies on the surface of the PANmod. The same is also proposed based on the suitability of the Freundlich isotherm for representing the adsorption data of Ni^2+^.

As in the case of the Temkin isotherm, both the Halsey and H–J models proved to fit better the adsorption data for Ni^2+^ compared to Pb^2+^, as reflected by the high *R^2^* values shown in Table 4. Based on the results of the Temkin model, the adsorption of Ni^2+^ on the PANmod occurred with a lower heat of adsorption (0.17 kJ mol^−1^) but a much higher equilibrium binding constant (288 L mg^−1^) compared to Pb^2+^ (Table 4). The suitability of both the Halsey and H–J models in describing the adsorption process relates the multilayer adsorption of Ni^2+^ on PANmod with its heteroporous nature, suggesting a heterogeneous pore distribution. Figure 6d illustrates the R–P model excellent fit of the adsorption data of both heavy metal ions; the high values of the *R^2^* in Table 4 confirm the same for the other three-parameter isotherms that were used, that is the Sips model, proposing both homogeneous and heterogeneous adsorption of the Ni^2+^ and Pb^2+^ ions on PANmod [45,46,47,48]. In describing the adsorption of Ni^2+^, the Sips model slightly overestimated the theoretical adsorption capacity compared to the experimental value; a lower energy of adsorption and lesser degree of heterogeneity was evaluated for Ni^2+^ in comparison to the adsorption of Pb^2+^.

## 4. Conclusions

Here, we studied the successful adsorption of the heavy metal ions Pb^2+^ and Ni^2+^ on an electrospun membrane of PAN nanofiber activated with NaHCO_3_ (PANmod). The FTIR analysis revealed the polar interactions between PAN nanofibers and NaHCO_3_, helping the successful adsorption of Ni^2+^ and Pb^2+^ on the PANmod surface. Furthermore, the FE-SEM images and the elemental analysis using EDX revealed the functionalization of the PAN nanofiber with NaHCO_3_, and the agglomeration of Pb^2^ and Ni^2+^ on PANmod.

After rapid uptake of the heavy metal ions by the PANmod during immediate contact (15 min), a linear increase in both the adsorption capacity and removal efficiency was observed; equilibrium was attained 60 min after the contact started. Among the conventional kinetic models, the PSO kinetic model describes best the adsorption of both ion types on the PANmod sample; the calculated *R^2^* values (0.9–0.99) closely agreed with the estimated adsorption capacities, and also with the experimental values.

The percentage removal, after increasing for both heavy metal ions from 2 to 7, decreased from 73% to 21% and from 91% to 73% for Pb^2+^ and Ni^2+^, respectively, as the solution pH increased further from 7.0 to 9.0. The uptake of Pb^2+^ and Ni^2+^ by the PANmod increased linearly as the initial concentration of metal ions increased from 5.0 to 20 mg L^−1^; at the maximum value of the initial ion concentration (50 mg L^−1^), the maximum adsorption capacities corresponding to the Pb^2+^ and Ni^2+^ ions were 13 and 56 mg g^−1^, respectively. The optimal dose of PANmod, corresponding to a maximum uptake of Pb^2+^ and Ni^2+^, was found to be 0.7 and 0.2 g, respectively; the adsorption capacity decreased with further increase in the adsorbent dose.

Among the two-parameter isotherm models, the Langmuir model fitted better the Pb^2+^ adsorption data, suggesting a monolayer adsorption. The Freundlich model fitted the Ni^2+^ adsorption on the surface of the PANmod better than that of Pb^2+^, reflecting a heterogeneous adsorption of Ni^2+^ with a uniform distribution of binding energies on the surface of the PANmod. The estimated values of the mean free energy of adsorption for the D–R isotherm, *E* < 8 kJ mol^−1^, reflected clearly a physical adsorption of both the heavy metal ions on the surface of the PANmod. As in the case of the Temkin isotherm, both the Halsey and H–J models proved to be better fits of the adsorption data for Ni^2+^ than that of to Pb^2+^, revealing the multilayer adsorption of Ni^2+^ onto PANmod with heteroporous nature and suggesting heterogeneous pore distribution.

## Figures and Tables

**Table 1:** Parameters of the nonlinear pseudo-first-order (PFO), pseudo-second-order (PSO), intraparticle diffusion of Weber and Morris (ID-WM), and Elovich kinetics models for the adsorption of Pb^2+^ and Ni^2+^ onto PANmod (Contact time = 0–300 min, PANmod dose = 0.5 g, pH = 5 ± 0.2 and 7 ± 0.2 for Pb^2+^ and Ni^2+^, respectively).

Kinetic Model	Parameter	Initial Metal Conc. (mg L^−1^)						
		Pb^2+^				Ni^2+^		
		5	10	15	20	10	15	20
	*q*_e exp_ (mg g^−1^)	6.24	10.07	12.51	12.67	21.36	29.43	37.41
PFO	*q_e cal_* (mg g^−1^)	6.11	9.73	12.21	12.24	20.68	28.5	36.51
	*k*_1_ (min^−1^)	0.13	0.13	0.14	0.099	0.31	0.31	0.29
	*R* ^2^	0.9	0.74	0.92	0.93	0.62	0.65	0.7
PSO	*q_e cal_* (mg g^−1^)	6.49	10.42	12.96	13.05	21.4	29.5	37.89
	*k*_2_ (g mg^−1^ min^−1^)	0.035	0.02	0.019	0.013	0.034	0.024	0.017
	*h* (mg g^−1^ min^−1^)	1.47	2.17	3.19	2.21	15.57	20.89	24.41
	*R* ^2^	0.98	0.94	0.99	0.9	0.94	0.95	0.98
ID-WM	*K_ip_* (mg g^−1^ min^1/2^)	0.15	0.27	0.28	0.33	0.21	0.31	0.45
	*C* (mg g^−1^)	4.41	6.78	9.07	8.18	18.53	25.38	32.05
	*R* ^2^	0.51	0.64	0.49	0.6	0.46	0.5	0.55
Elovich	*α* (mg g^−1^ min^−1^)	70.69	65.3	241.7	41.1	2.31 × 10^7^	1.45 × 10^7^	3.57 × 10^6^
	*β* (g mg^−1^)	1.51	0.88	0.8	0.66	1.03	0.72	0.51
	*R* ^2^	0.79	0.89	0.77	0.68	0.74	0.78	0.81

**Table 2:** Comparison of adsorption capacities of PANmod for Pb^2+^ and Ni^2+^ with other similar adsorbents.

Adsorbent	Contaminant	Maximum Adsorption Capacity, mg g^−1^	Reference
PAN_mod_	Pb^2+^	15	This study
PAN_mod_	Ni^2+^	60	This study
Polyaniline grafted chitosan	Pb^2+^	16	[36]
APAN nanofiber mat	Pb^2+^	60	[37]
PAN/SiO_2_ composite nanofiber	Ni^2+^	138.7	[38]
ACNFs PAN/ZnO	Pb^2+^	120	[39]
PAN–TiO_2_–APTES	Ni^2+^	147	[40]
P-PAN fibers	Pb^2+^	72.5	[41]

**Table 3:** Mathematical expressions of the nonlinear and linearized two-parameter isotherm models.

Isotherm	Nonlinear	Linearized
Langmuir	$q_{e}=\frac{q_{m}K_{L}C_{e}}{\left(1+K_{L}C_{e}\right)}\;$ *R_L_* = (1*+K_L_C_0_*) ^−1^	$\frac{1}{q_{e}}=\frac{1}{q_{m}}+\left(\frac{1}{q_{m}K_{L}}\right)\frac{1}{C_{e}}$
Freundlich	qe=KFce1n	$logq_{e}=logK_{F}+\frac{1}{n}logC_{e}$
Dubinin–Radus-Kevich	$q_{e}=q_{m}\exp\left(-K_{DR}\varepsilon^{2}\right)$ $\varepsilon =RT\;\ln\left(1+\frac{1}{C_{e}}\right)$	$\ln q_{e}=\ln q_{m\;}-K_{DR}\varepsilon^{2}$ *E* = $\frac{1}{\sqrt{2K_{DR}}}$
Halsey	$q_{e}=\exp\left(\frac{\ln k_{H\;}-\ln C_{e}}{n_{H}}\right)$	$\ln q_{e=}\frac{1}{n_{H}}\;\ln\;k_{H\;}-\frac{1}{n_{H}}\;\ln\;C_{e}$
Temkin	$q_{e}=\frac{RT}{H_{ads}}\ln\;(K_{T}\;C_{e})$	$q_{e}=\frac{RT}{b_{T}}\ln\;A_{T}+\frac{RT}{b_{T}}\ln\;C_{e}$
Harkins–Jura	$q_{e}={\left(\frac{A_{HJ}}{B_{HJ}-logC_{e}}\right)}^{\frac{1}{2}}$	$\frac{1}{q_{e}^{2}}=\left(\frac{B_{HJ}}{A_{HJ}}\right)-\left(\frac{1}{A_{HJ}}\right)\;logC_{e}$

**Table 4:** Estimated parameter values in the nonlinear and linearized isotherm models for Pb^2+^ and Ni^2+^ (contact time = 60 min, PANmod dose= 0.5 g, pH = 5 ± 0.2 and 7 ± 0.2 for Pb^2+^ and Ni^2+^, respectively).

Isotherm	Parameter	Pb^2+^		Ni^2+^	
		Nonlinear	Linear	Nonlinear	Linear
	*q*_e exp_, mg g^−1^	13.05 (against 30 mg L^−1^)		56 (against 50 mg L^−1^)	
Langmuir	*q_m_*_,_ mg g^−1^	13.67	14.12	49.38	44.25
	*K_L_*_,_ L mg^−1^	0.56	0.44	2.66	11.89
	*R_L_*	0.056	0.07	0.0075	0.002
	*R* ^2^	0.91	0.96	0.62	0.78
Freundlich	*q_m_*, mg g^−1^	12.90	13.75	63.44	64.22
	*K_F_*, ((mg g^−1^)(L mg^−1^)^1/n^)	7.74	7.20	31.37	33.44
	*n*	6.67	5.26	5.56	6.00
	*R* ^2^	0.64	0.72	0.97	0.97
D–R	*q_m_*_,_ mg g^−1^	12.74	12.7	48.55	44.54
	*K_DR_*, (mol kJ^−1^)^2^	66 × 10^−8^	60 × 10^−8^	15 × 10^−8^	2 × 10^−8^
	*E*, kJ mol^−1^	0.87	0.91	1.8	5.0
	*R* ^2^	0.96	0.98	0.31	0.66
Halsey	*q*_e cal_, mg g^−1^	12.3	11.02	54.15	62.3
	*n_H_*	−6.7	−5.26	−5.7	−6.00
	*K_H_*	0.000	0.65	0.000	0.177
	*R* ^2^	0.64	0.72	0.97	0.97
Temkin	*K_T_*_,_ L mg^−1^	45.20	45.09	278.57	278.58
	*H_ads_*_,_ kJ mol^−1^	0.56	1.4	0.17	0.4
	*R* ^2^	0.71	0.74	0.92	0.97
H–J	*A_HJ_*, mg g^−1^	76.9	89.29	175	1428.6
	*B_HJ_*	4.5	1.96	2.9	1.71
	*R* ^2^	0.63	0.65	1.00	0.95
Sips	*q_m_*_,_ mg g^−1^	12.87		64.81	
	*K_S_*, L g^−1^	0.29		0.5	
	*n_S_*	0.76		0.18	
	*R* ^2^	0.97		0.99	
R–P	*K_RP_*_,_ L g^−1^	4.73		7.1	
	*α*, L mg^−1^	0.2		2.25	
	*β*	0.15		0.82	
	*R* ^2^	0.97		0.99	

## Data Availability

Data is contained within the article.
